# Intubation success in prehospital emergency anaesthesia: a retrospective observational analysis of the Inter-Changeable Operator Model (ICOM)

**DOI:** 10.1186/s13049-022-01032-2

**Published:** 2022-07-08

**Authors:** James Price, Kate Lachowycz, Alistair Steel, Lyle Moncur, Rob Major, Ed B. G. Barnard

**Affiliations:** 1 Department of Research, Audit, Innovation, and Development (RAID), East Anglian Air Ambulance, Helimed House, Hangar 14, Gambling Close, Norwich, UK; 2 Emergency Department, Cambridge University Hospitals NHS Foundation Trust, Cambridge, UK; 3 Magpas Air Ambulance, Centenary House, St Mary’s Street, Huntingdon, UK; 4 The Queen Elizabeth Hospital King’s Lynn NHS Foundation Trust, King’s Lynn, UK; 5 Essex and Herts Air Ambulance, North Weald Airfield, Merlin Way, North Weald Bassett, Epping, CM16 6HR UK; 6 Academic Department of Military Emergency Medicine, Royal Centre for Defence Medicine (Research and Clinical Innovation), Birmingham, UK

**Keywords:** Pre hospital care, HEMS, RSI, PHEA, Intubation

## Abstract

**Background:**

Pre hospital emergency anaesthesia (PHEA) is a complex procedure with significant risks. First-pass intubation success (FPS) is recommended as a quality indicator in pre hospital advanced airway management. Previous data demonstrating significantly lower FPS by non-physicians does not distinguish between non-physicians operating in isolation or within physician teams. In several UK HEMS, the role of the intubating provider is interchangeable between the physician and critical care paramedic—termed the Inter-Changeable Operator Model (ICOM). The objectives of this study were to compare first-pass intubation success rate between physicians and critical care paramedics (CCP) in a large regional, multi-organisational dataset of trauma PHEA patients, and to report the application of the ICOM.

**Methods:**

A retrospective observational study of consecutive trauma patients ≥ 16 years old who underwent PHEA at two different ICOM Helicopter Emergency Medical Services in the East of England, 2015–2020. Data are presented as number (percentage) and median [inter-quartile range]. Fisher’s exact test was used to compare proportions, reported as odds ratio (OR (95% confidence interval, 95% CI)), *p* value. The study design complied with the STROBE (Strengthening The Reporting of Observational studies in Epidemiology) reporting guidelines.

**Results:**

In the study period, 13,654 patients were attended. 674 (4.9%) trauma patients ≥ 16 years old who underwent PHEA were included in the final analysis: the median age was 44 [28–63] years old, and 502 (74.5%) were male. There was no significant difference in the FPS rate between physicians and CCPs—90.2% and 87.4% respectively, OR 1.3 (95% CI 0.7–2.5), *p* = 0.38. The cumulative first, second, third, and fourth-pass intubation success rates were 89.6%, 98.7%, 99.7%, and 100%. Patients who had a physician-operated initial intubation attempt weighed more and had a higher heart rate, compared to those who had a CCP-operated initial attempt.

**Conclusion:**

In an ICOM setting, we demonstrated 100% intubation success in adult trauma patients undergoing PHEA. There was no significant difference in first-pass intubation success between physicians and CCPs.

## Background

Prehospital airway management with rapid sequence induction (RSI) of anaesthesia is a necessary and potentially lifesaving intervention for a substantial proportion of severely injured trauma patients [1]. Compared to in-hospital RSI, prehospital emergency anaesthesia (PHEA) is a complex procedure with significant risks and additional environmental and clinical challenges [2]. Complications such as hypoxia, hypotension, aspiration, and misplaced endotracheal tubes are reported even within high-volume and well-governed physician-paramedic helicopter emergency medical services (HEMS) [1]. Therefore, PHEA is associated with a significant risk of potentially-avoidable morbidity and mortality [3].

In recognition of the relationship between the incidence of PHEA complications and the number of intubation attempts [4], the first-pass intubation success (FPS) rate, defined as the rate of successful tracheal intubation at first attempt, is a recommended key performance indicator and quality marker [5]. Current United Kingdom (UK) guidance advocates that the first intubation attempt is performed by the most experienced anaesthesia provider, usually the physician within the team [6]. Meta-analyses have demonstrated a significantly higher intubation success rate for physicians compared to non-physicians [7, 8]. However, these studies do not differentiate between non-physician providers (typically paramedics and nurses) intubating in isolation or alongside prehospital physicians.

In several UK HEMS, the role of the intubating provider is interchangeable between the physician and critical care paramedic—termed the Inter-Changeable Operator Model (ICOM). There are several theoretical advantages to ICOM, including an additional airway specialist on scene, an airway assistant with an intimate practical knowledge of the procedure, and the ability for either clinician within the team to ‘rescue’ a potentially failed intubation attempt. These systems do not stipulate which team member should be the initial operator, and protocols include a change of operator between cases, and after one or two failed intubation attempts. Some UK HEMS have reported small data series that suggest there is no significant difference in first-pass intubation success rates between physicians and non-physicians in the ICOM setting [9–11]. The objective of this study was to compare first-pass intubation success rate between physicians and critical care paramedics (CCP) in a large regional, multi-organisational dataset of trauma PHEA patients, and to report the application of the ICOM.

## Methods

### Emergency medical service

The East of England is a geographic area of 20,000 km^2^, containing a population of approximately 6.4 million people [12]. The East of England Ambulance Service NHS Trust (EEAST) provides the statutory Emergency Medical Service (EMS) response in this region, and has been previously described [13]. The EMS response can be augmented by one of five physician-CCP HEMS teams; dispatched at the discretion of the CCP-led critical care desk at one of the EEAST Emergency Operation Centres [14].

### HEMS teams

The core of each team consists of a physician and a CCP with at least three years’ post-registration experience. However, to facilitate education and training, a clinical supervisor frequently accompanies the core team. Supervisors are most often a senior physician, but when training a new CCP this role is undertaken by a senior CCP [15]. Physicians in these teams are predominantly emergency medicine (EM) or anaesthesia consultants or senior registrars, with a minimum of six months training in hospital anaesthesia and extensive experience in the management of acutely unwell and injured patients. Prior to independent practice, physicians undergo further specialist training in pre hospital care, including a period of supervised practice and a local formative assessment by a pre hospital care consultant. Within ICOM systems, the decision as to who will perform laryngoscopy is typically decided before scene arrival, according to training needs, experience, team dynamics, and previous missions. Three of the five HEMS in the East of England utilise ICOM; two are operated by East Anglian Air Ambulance (EAAA) and one is operated by Magpas Air Ambulance (Magpas). Both services are classified as high-volume HEMS (> 50 PHEA cases per annum) [16], and have similar operating models that have been previously described [11, 14].

Both organisations deliver PHEA according to a shared standard operating procedure. This includes a standardised drug regime (ketamine 1–2 mg/kg, rocuronium 1 mg/kg, ± fentanyl) [17]. Typically intubation was performed using direct laryngoscopy. From 2017, the option (for use at the discretion of the operating clinician) of videolaryngoscopy was introduced (McGrath® videolaryngoscope, Aircraft Medical, Edinburgh, UK). Standard practice does not include the routine application of cricoid pressure, and if required, neck immobilization is performed with manual inline stabilization with an open/absent cervical collar. Both services use the HEMSbase electronic medical record software (MedicOne Systems Ltd, UK).

### Inclusion criteria

In this retrospective observational study, a consecutive sample of trauma patients (as recorded in HEMSbase) ≥ 16 years old, attended to by EAAA (1st January 2015 to 31st December 2020) or Magpas (1st November 2015 to 31st December 2020, owing to later implementation of HEMSbase), and who underwent PHEA (defined as drug-assisted intubation) were included.

### Exclusion criteria

Each clinical record was reviewed by one of the study authors to identify exclusions, which included: unascertainable patient age, secondary transfer cases, duplicate cases, patients intubated in arrest, intubation by a non-HEMS team provider, and mechanisms not meeting the definition of trauma (injury through the transfer of kinetic energy); including medical cases initially coded as ‘trauma’, overdose, hanging, asphyxiation, burns, drowning, electrocution (and similar non-trauma mechanisms).

### Primary outcome

The first-pass intubation success rate of physicians compared to CCPs.

### Secondary outcomes

Secondary outcomes were: (1) report the cumulative first, second, third, and fourth-pass intubation success rates; (2) compare the pre-PHEA physiology and characteristics likely to affect intubation success in patients whose initial intubation attempt was undertaken by a physician compared to a CCP; (3) report the proportion of initial intubation attempts and FPS rate by professional group (CCP, EM physicians, anaesthesia physicians); (4) compare the crossover rate between professional groups. The crossover rate concerns the cohort of patients with a failed initial intubation attempt and was calculated as the proportion of cases in which the professional group of the final successful intubation was different to that of the initial unsuccessful attempt.

### Data collection

Data were extracted from HEMSbase at both organisations, and included: patient demographics (age, sex, estimated weight—as a surrogate for body mass index owing to the absence of patient height data), whether the patient was trapped on the arrival of HEMS (e.g. in a damaged vehicle), PHEA (HEMS team members and role, number of intubation attempts, provider professional group), and pre-PHEA physiological variables (heart rate (HR), respiratory rate (RR), systolic blood pressure (SBP), diastolic blood pressure (DBP), and shock index (SI); HR divided by SBP). Combined data were collated into a single data sheet in Excel (Microsoft Corporation (2021), *Microsoft® Excel for Mac, Version 16.45*), and stored on a secure EAAA server.

Physiological variables were captured from time-calibrated patient monitors (EAAA—X Series, ZOLL Medical Corporation, Runcorn, UK; Magpas—Tempus Pro, Philips Electronics UK Ltd, Farnborough, UK) as part of standard patient care, and uploaded automatically to HEMSbase. Physiological data from each case was reviewed by one of the study authors from the HEMSbase output, and overtly erroneous entries were deleted. If the data was equivocal, a decision to include/exclude was reached by consensus of three authors (JP, KL, EB) after independent review of all available case notes.

### Ethical review

This study met the UK National Institute for Health Research criteria for a service evaluation. All data used for this study are routinely collected as part of standard prehospital data collection; formal ethical approval was therefore waived. The study was approved and registered with the EAAA Department of Research, Audit, Innovation & Development (EAAA 2021/001) and the Magpas Air Ambulance Clinical Governance Group. The study design complied with the STROBE (Strengthening the Reporting of Observational studies in Epidemiology) reporting guidelines [18].

### Data analysis

Data have been reported as number (percentage), number (percentage (95% confidence interval (95% CI), Wilson/Brown method)), and mean (± standard deviation) or median [interquartile range] as appropriate. Fisher’s exact test has been used to compare two proportions, and is reported with a Baptista-Pike calculated odds ratio (OR) with 95% CI (Wilson/Brown method) and a *p* value; three-way analysis of proportions has been analysed with a Chi-square test and is reported as a *p* value. Normally distributed data have been compared with an unpaired, two-tailed Student’s t test (with Welch’s correction for unequal standard deviations) and reported as a *p* value. Non-normally distributed data have been compared with a Mann–Whitney U test and reported as a *p* value. Statistical analyses were performed in Prism for MacOS (v.9.2.0, GraphPad Software, San Diego, CA, USA), using the software’s recommended analyses; significance was pre-defined at < 0.05 and no corrections were made for multiple comparisons.

## Results

**Fig. 1:**
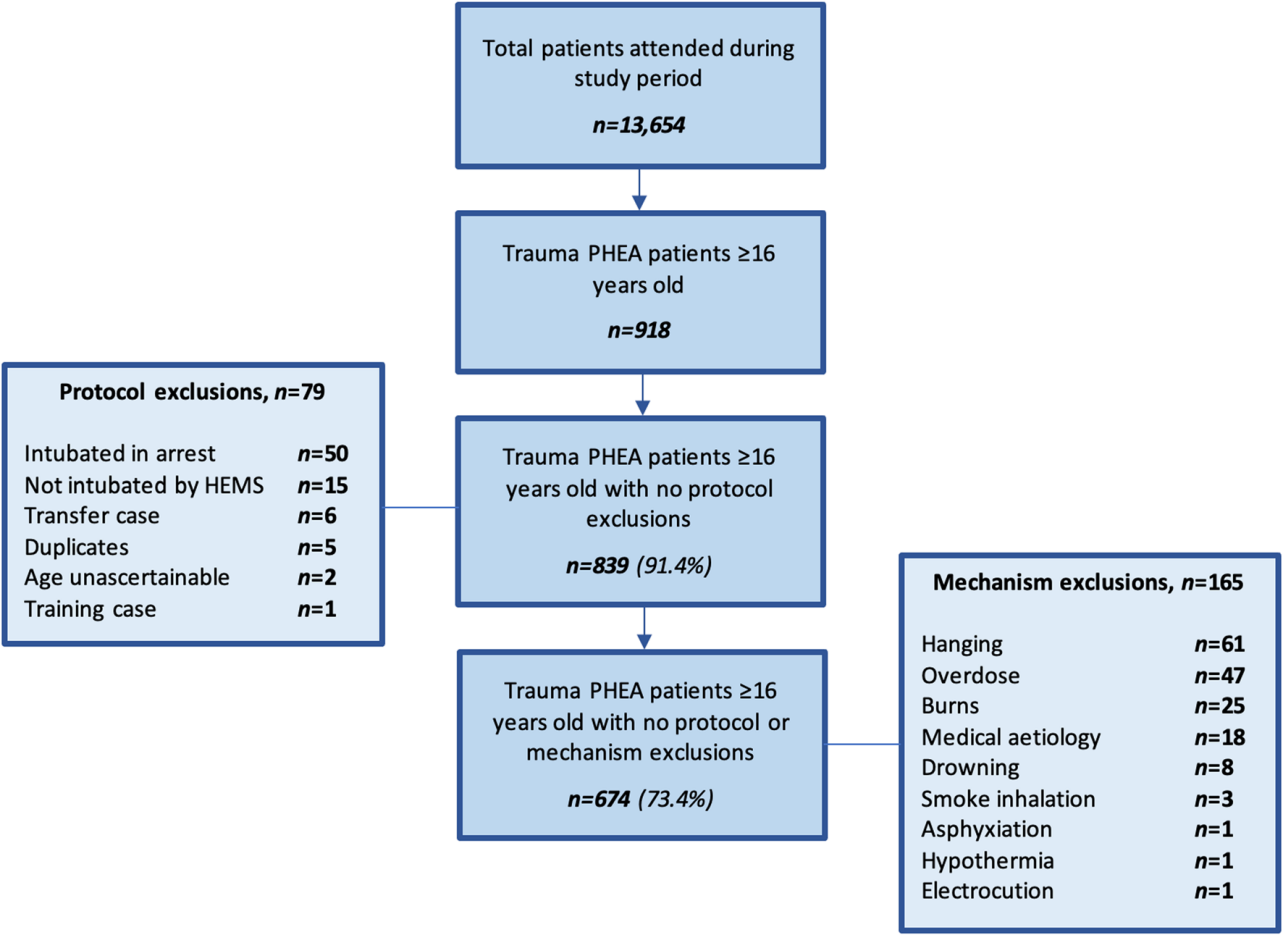
Study inclusion and exclusion criteria. *PHEA* prehospital emergency anaesthesia, *HEMS* helicopter emergency medical service

During the study period, 13,654 patients were attended (EAAA *n* = 9,528 and Magpas *n* = 4,126). 918 trauma patients ≥ 16 years old underwent PHEA and were recorded in HEMSbase. Following 79 protocol exclusions and a further 165 non-trauma mechanism exclusions, 674 trauma PHEA cases were included in the final analysis (*n* = 485 (72.0%) EAAA, and *n* = 189 (28.0%) Magpas). The intubating provider was identifiable in all remaining cases, and there were no cases in which the number of attempts at intubation was not reported, Fig. 1.

The median age was 44 [28–63] years old, and 502 (74.5%) were male. The most prevalent mechanism of injury was road traffic collision (*n* = 388, 56.8%), followed by fall (*n* = 203, 30.1%), intentional self-harm (*n* = 24, 3.6%), sporting incident (*n* = 23, 3.4%), assault (*n* = 21, 3.1%), and other (*n* = 20, 3.0%). All 674 patients were successfully intubated prehospital, Table 1.

**Table 1:** Patient demographics and pre-PHEA physiological variables, presented as a total cohort (n = 674), those with a physician and those with a critical care paramedic as the initial operator

Variable	Overall	Physician initial operator	CCP initial operator	*p* value
Patients (n (%))	674	571 (84.7%)	103 (15.3%)	< 0.0001***
Age (years)	44.0 [28.0–63.0]	44.0 [28.5–62.0]	40.0 [27.8–64.8]	0.94
Estimated weight (kg)	75.0 [70.0–80.0]	80.0 [70.0–85.0]	70.0 [70.0–80.0]	< 0.05*
Male sex (n (%))	502 (74.5%)	426 (74.6%)	76 (73.8%)	0.90
Entrapped (n (%))	100 (14.8%)	90 (15.8%)	10 (9.7%)	0.13
*Pre-PHEA variables*				
HR (beats/min)	99.7 (± 29.0)	101.0 (± 29.4)	91.5 (± 25.0)	< 0.0001***
SBP (mmHg)	134.4 (± 35.1)	134.0 (± 34.7)	137.2 (± 37.3)	0.48
MAP (mmHg)	100.3 (26.1)	100.0 (± 26.4)	101.9 (± 24.4)	0.58
RR (breaths/min)	22.1 (± 14.1)	22.2 (± 11.9)	21.6 (± 23.5)	0.83
Shock Index	0.81 (± 0.45)	0.83 (± 0.47)	0.70 (± 0.26)	< 0.0001***
*Non-entrapped patients*				
Patients (n (%))	574	481 (83.8%)	93 (16.2%)	
Arrive to PHEA (min)	19.0 [14.0–26.0]	20.0 [14.0–27.0]	18.0 [12.0–24.0]	< 0.05*

*CCP* critical care paramedic, *PHEA* prehospital emergency anaesthesia, *HR* heart rate, *SBP* systolic blood pressure, *MAP* mean arterial pressure, *RR* respiratory rate. The shock index was calculated as HR/SBP. ‘Arrive to PHEA’ is the time in minutes from the HEMS team arrival on scene until the time of PHEA

### Primary outcome

There was no significant difference in FPS rate between physicians and CCPs—90.2% and 87.4% respectively—OR 1.3 (95% CI 0.7–2.5), *p* = 0.38.

### Secondary outcomes

#### Intubation success rates

Overall, the cumulative first, second, and third-pass intubation success rates were 89.6%, 98.7%, and 99.7% respectively; *n* = 2 (0.3%) patients were intubated on a fourth attempt, Table 2.

**Table 2:** Intubation success rates for each number of attempts, presented as individual increments and cumulative totals (*n* = 674)

Number of attempts required to pass an endotracheal tube	Success/*n*	Success/*n* (cumulative)	Cumulative success/% (95% CI)
One	604	604	89.6% (87.1–91.7)
Two	61	665	98.7% (97.5–98.7)
Three	7	672	99.7% (98.9–100.0)
Four	2	674	100.0% (99.4–100.0)

*n* number, *95% CI* 95% CI confidence interval (Wilson/Brown method)

Both cases requiring four attempts at intubation were complicated. The first included two failed videolaryngoscopic intubation attempts (good view, unable to pass the bougie/tube) by the physician, an attempt by the CCP, and a final successful attempt with direct laryngoscopy by the supervising EM physician. The second case was a patient with no recordable blood pressure and agonal respirations—there was no end-tidal carbon dioxide trace after the first attempt by the physician, so the endotracheal tube was removed (concern for oesophageal intubation), the second attempt was with an AirTraq® optical laryngoscope by the same provider and was unsuccessful, the third attempt was by the CCP with direct laryngoscopy, and the fourth successful attempt was by the initial operating physician with an AirTraq® loaded with a bougie.

#### Pre-PHEA physiology and characteristics

Cases with a physician and CCP initial operator had similar age and gender proportions. However, patients who weighed more, had a higher heart rate and shock index were more likely to undergo physician-operated initial intubation attempts, Table 1.

Overall, entrapped patients had a longer interval between HEMS team arrival and PHEA compared to those non-entrapped—28.0 [19.0–37.0] minutes compared to 19.0 [14.0–26.0] minutes, *p* < 0.0001. Therefore, non-entrapped patients were used to compare arrival to PHEA time between CCPs and physicians. We observed that patients with a first intubation attempt by a physician had a longer duration from HEMS team arrival to PHEA compared to CCPs, Table 1.

#### Physician compared to CCP intubations

The first intubation attempt was performed more frequently by a physician—OR 30.7 (95% CI 22.8–41.4), *p* < 0.0001, Table 1. A total of n = 8 patients received an initial intubation attempt from a General Practitioner (GP) physician; n = 6 (75.0%) were successful, and owing to the low incidence and risk of type-2 error, cases with a GP as the team physician (n = 11/674, 1.6%) were excluded from further secondary analyses. In the remaining n = 663 patients, the highest proportion of initial operators were EM physicians, followed by CCPs and anaesthesia physicians, Table 3.

**Table 3:** The number and proportion of initial operators and first-pass intubation success rates by professional group (n = 663)

	CCP	Physician—EM	Physician—Anaes	Chi-square *p* value
Initial operator n (%)	100 (15.1%)	473 (71.3%)	90 (13.6%)	–
First-pass success n (%)	90 (90.0%)	427 (90.3%)	81 (90.0%)	0.99

*CCP* critical care paramedic, *EM* emergency medicine, *Anaes* anaesthesia

We observed no significant difference in FPS rate between physicians of either an EM or anaesthesia background, and CCPs—OR 1.0 (95% CI 0.5–2.1) *p* > 0.99. Further analysis of primary operator revealed that EM physicians were significantly more likely than anaesthesia physicians to undertake the first attempt at laryngoscopy (rather than their respective CCP team member)—473/528 (90.0%) and 90/135 (67.9%), OR 4.3 (95% CI 2.7–6.7), *p* < 0.0001.

#### Crossover analysis

Sixty-eight patients required more than one intubation attempt (excluding those with a GP physician in the team). In *n* = 51 (75.0%), the initial and final successful intubator were the same individual provider (*n* = 46 two attempts, *n* = 4 three attempts, *n* = 1 four attempts). In the remaining 17 cases, the first and final intubators were different—all had an identifiable team role: CCP, physician operator, or physician supervisor.

There was a trend of higher crossover in the CCP initial operator cohort (4/13, 30.8%) compared to physician initial operator cohort (13/55, 23.6%), but this did not reach significance—OR 1.4 (95% CI 0.4–5.1) *p* = 0.72. However, in the physician initial operator cohort, we observed no instances of crossover in failed first-pass intubation attempts by anaesthesia physicians: total of *n* = 9, of which *n* = 1 required three attempts at intubation by the same clinician.

## Discussion

In this study, we observed a 100% intubation success rate in a large cohort of adult trauma patients undergoing PHEA, with no requirement for either rescue supraglottic airway devices or surgical airways. Contrary to a recent meta-analysis, we demonstrated that in an ICOM setting there was no significant difference in first-pass success between physicians and CCPs. There was a signal that patients who had a physician-operated initial attempt were more unwell. We also observed that physicians were much more likely than CCPs to undertake the initial attempt at intubation and that there was a trend of CCPs being more likely to hand over the second intubation to a physician (compared to the other way around). These data further support the safe practice and delivery of prehospital anaesthesia in the UK when performed in well-governed, high-volume HEMS [16].

The primary objective in PHEA is to achieve first-pass intubation success. Current national guidance suggests the clinician with the most anaesthetic experience take on the role of primary operator, usually the physician, in the traditional ‘physician-paramedic’ HEMS model [6]. HEMS operating the ICOM do not stipulate the individual allocated to the role of ‘operator’ for intubation, and indeed advocate CCP development by encouraging paramedics to take on the operator role. In 2013, von Volpelius-Feldt et al. first described the ICOM in UK HEMS [10]; successfully demonstrating the ability of both physicians and CCPs to deliver effective PHEA, when extensive theoretical and practical joint training is delivered in systems with robust clinical governance. McQueen et al. further supported the ICOM, demonstrating a 0% failed intubation rate for CCPs during PHEA, and a superior first-pass success rate compared to physicians; 94.3% and 88.0% respectively [9]. However, the authors acknowledged the limited sample size and the inability to determine the factors associated with patient selection and therefore the choice of operator, concluding that CCPs may potentially be deferring cases with anticipated difficulties to physicians. In our study, we further support the implementation of the ICOM within UK HEMS, demonstrating intubation success rates equivalent to other systems where intubation is performed solely by physicians [19], and CCP intubation success rates in excess of those previously reported in large prehospital intubation meta-analyses [7, 8]. Whilst there are no national guidelines on the acceptable first and second pass success rates for PHEA in trauma, the 2021 European Resuscitation Council Advanced Life Support guideline defines a high success rate as intubation within two attempts 95% of the time [20]; our data (including the lower bound of the 95% CI) exceeds this standard.

In this study, there was a trend of a higher proportion of entrapped patients in the physician initial operator cohort, but this did not reach significance. We found that in non-entrapped patients there was a significantly longer interval between HEMS arrival and PHEA in the physician-operated cohort. Furthermore, patients who weighed more and those who had a higher heart rate and shock index (as a product of heart rate rather than a difference in systolic blood pressure), were statistically more likely to undergo initial physician-operated intubation attempts. These data suggest that in patients with perceived anatomical or physiologically difficult airways [21], the initial operator role is more likely to be deferred to the physician. However, the longer time to PHEA in the initial physician operator cohort may be confounded by more unwell patients, potentially requiring more thorough pre-anaesthetic optimization or additional life-saving interventions [22].

The professional group with the highest FPS rate was EM physicians. We did not find a significant difference in first-pass success rate between physicians of either an EM or anaesthesia background, or CCPs, contrasting results to previous work, where anaesthesia specialists have a higher prehospital intubation success rate [8, 19, 23]. The comparable EM physician success rate in our study may partly be due to the standardization of experience required to undertake PHEA (a minimum requirement for physicians to have completed six months of anaesthesia training) [6], or potentially due to the introduction of a national PHEM training programme that provides regular structured training opportunities and enhanced supervised practice [24]. The drive to undertake PHEA as an identifiable clinical skill in the national PHEM training programme may also explain the very high proportion of first intubation attempts by physicians compared to CCPs in this ICOM setting. However, further analysis of our data does demonstrate that anaesthesia physicians were more likely than EM physicians to defer the first intubation attempt to their CCP team member. Logically, this is likely to be due to the relative comfort of anaesthetists to supervise intubation, and the drive of EM physicians to maintain their airway skills, exposure to which overall is likely to be less frequent than their anaesthesia colleagues.

In a small but significant proportion of patients, more than one attempt was required for successful intubation*.* The shared PHEA standard operating procedure (SOP) at both organisations state that further attempts at intubation should only be undertaken following an improvement in intubating conditions [15]. If no patient or operator characteristics can be identified, a change of operator is encouraged. In n = 17/663, 2.6% of the cases in the crossover analysis that required more than one intubation attempt, the first and final operators were different. A potential benefit of HEMS teams training and operating within the ICOM system is that both clinicians are competent airway providers, with equal training opportunities, governance, exposure, and prehospital experience. Theoretically, this model has the greatest utility in the first few months of newly-qualified HEMS physicians operating, but we were unable to test this hypothesis with these data.

On further analysis, we found that only physicians with an EM background engage with crossover. In all cases of failed initial attempts by physicians from an anaesthesia background, we observed no instances of crossover, with the same physician persisting until achieving success on a second or subsequent intubation attempt. However, the small number of cases renders this observation inconclusive. A change of operator is a recognised successful strategy for failed first-pass intubation in both hospital and prehospital practice [25, 26]. Physicians from an anaesthetic background recognise that they are likely to have significantly more advanced airway management skills [27], and therefore switching to a ‘less experienced’ non-anaesthetist operator may be perceived to delay definitive airway management, especially as the initial anaesthetic operator may be best placed to perform subsequent attempts. Further qualitative work is required to further understand this observation.

We acknowledge that crossover occurred in only a very small proportion (2.6%) of all patients undergoing PHEA in this cohort. Whilst this number may appear low and therefore potentially defuncts the ICOM concept, it should be appreciated that airway emergencies such as the inability to intubate or ventilate a patient after the administration of muscle relaxant may result in significant complications or even death [28]. Given this, one in 40 rescued airway attempts by an alternative competent provider at scene, is not an insignificant figure, may further demonstrate an advantage of the ICOM model over physician-only delivered PHEA, and might explain the exceptionally high intubation success rate in this study.

### Limitations

This is a retrospective study of intubation success rates in PHEA. Non-physiological data in the HEMSbase electronic medical record is self-reported by the clinical team immediately following patient care, and is therefore subject to both recall and reporting biases. From the available data, we cannot be confident that all patient and/or scene factors influencing the decision as to who will be first to perform laryngoscopy have been captured. This is likely to vary significantly based on the experience of each clinician within the team, and additional prospective work is required to further understand this complex decision-making process.

The assessment of complication rates including the incidence of post-intubation hypoxia and hypotension, and the correlation between the number of intubation attempts and patient outcome, were beyond the scope of this observational study. Owing to the high overall first-pass success rates in this study, we acknowledge the findings in our sub-group analyses may be susceptible to a type-2 error due to the limited sample size. Furthermore, we were not able to control for new team members rotating through the system during the study period with varied prior clinical experience. However, all providers are enrolled in a regional induction programme, familiarized with SOPs, and undergo a formal assessment process before independent team practice.

## Conclusions

In an ICOM setting, we demonstrated 100% intubation success in adult trauma patients undergoing PHEA. There was no significant difference in first-pass intubation success between physicians and CCPs.

## Data Availability

The datasets used and/or analysed during the current study are available from the corresponding author on reasonable request.

## Abbreviations

CCP: Critical care paramedic
DBP: Diastolic blood pressure
EAAA: East Anglian Air Ambulance
EEAST: East of England Ambulance Service
EM: Emergency medicine
EMS: Emergency medical service
FPS: First-pass intubation success
GP: General Practitioner
HEMS: Helicopter Emergency Medical Service
HR: Heart rate
ICOM: Inter-changeable operator model
MAP: Mean arterial pressure
OR: Odds ratio
PHEA: Pre hospital emergency anaesthesia
RR: Respiratory rate
RSI: Rapid sequence induction
SBP: Systolic blood pressure
SI: Shock index
UK: United Kingdom
